# The role of anticipated regret in choosing for others

**DOI:** 10.1038/s41598-021-91635-z

**Published:** 2021-06-15

**Authors:** Shiro Kumano, Antonia Hamilton, Bahador Bahrami

**Affiliations:** 1 Institute of Cognitive Neuroscience, University College London, Alexandra House, 17 Queen Square, London, WC1N 3AZ UK; 2 NTT Communication Science Laboratories, Nippon Telegraph and Telephone Corporation, 3-1 Morinosato-Wakamiya, Atsugi, Kanagawa 243-0198 Japan; 3 Faculty of Psychology and Educational Sciences, Ludwig Maximilian University, Leopoldstrasse 13, 80802 Munich, Germany; 4 Department of Psychology, Royal Holloway University of London, Egham, TW20 0EX Surrey UK; 5 Centre for Adaptive Rationality, Max Planck Institute for Human Development, Lentzeallee 94, 14197 Berlin, Germany

**Keywords:** Human behaviour, Social behaviour, Decision

## Abstract

In everyday life, people sometimes find themselves making decisions on behalf of others, taking risks on another’s behalf, accepting the responsibility for these choices and possibly suffering regret for what they could have done differently. Previous research has extensively studied how people deal with risk when making decisions for others or when being observed by others. Here, we asked whether making decisions for present others is affected by regret avoidance. We studied value-based decision making under uncertainty, manipulating both whether decisions benefited the participant or a partner (beneficiary effect) and whether the partner watched the participant’s choices (audience effect) and their factual and counterfactual outcomes. Computational behavioural analysis revealed that participants were less mindful of regret (and more strongly driven by bigger risks) when choosing for others vs for themselves. Conversely, they chose more conservatively (regarding both regret and risk) when being watched vs alone. The effects of beneficiary and audience on anticipated regret counteracted each other, suggesting that participants’ financial and reputational interests impacted the feeling of regret independently.

## Introduction

We frequently make decisions for others. The change of viewpoint matters. For example, people display greater wisdom in contemplating the same problem when it has been framed as somebody else’s problem versus when framed as their own^1^. In everyday life, making decisions for others is often complicated by their presence. For example, choosing the wine for a group of friends in a restaurant would be stressful enough (and we may end up regretting having accepted to order for the table) but, many of us would find it even more difficult if the guests were to stop their conversation to hear your verdict. Our study looks precisely at the intersection of these two very common everyday life experiences: the role of anticipated regret in choosing for others (versus ourselves) in their presence (vs absence).

## The role of regret in deciding for others

Numerous studies have looked at changes in risk attitude when participants are making decisions for others versus for themselves. Stone et al.^2^ failed to find support for a meaningful difference in risk taking for self-versus for others when asking people to choose the lottery that they would want to play for themselves versus for an imaginary (absent) or real (present) other person. Since Stone et al.’s study^2^, many have produced evidence for reduced risk aversion when making decisions for others (see^3^). Mengarelli et al.^4^ showed in three different but related risk attitude tasks that subjects are less loss averse for others. Zhang et al.’s findings that framing effects were stronger for self and close other^5^ added some complexity to the picture. More risk aversion under gain frames and more risk seeking under loss frame were observed for decisions for self but these effects were weaker when the beneficiary of the decision was a stranger. Hsee and Weber^6^ argued that risk attitudes differ for self and other because decision makers may assume that others have a different risk attitude. They found that people made less risky choices for themselves than what they predicted abstract others would make for them. The results suggested that abstract others are assumed to be more risk taking.

Another reason which could explain the different risk attitudes for self and other is that people may try to achieve different goals when making decisions for themselves versus for others. Polman^7^ showed that when we buy a (for example) sweater for ourselves, we might worry more about getting the wrong colour (i.e. an error of commission). When we buy a sweater for our father, however, we may worry about missing out an even better sweater that we would have found if we had looked more (i.e. error of omission). Polman^7^ argued that making decisions for self is motivated by a *prevention* focus, aiming to minimise errors of commission (bad outcome occurring). On the other hand, a proxy decision maker is motivated, Polman argued, by a *promotion* focus and aims to minimise errors of omission (good outcomes failing to occur).

Supporting the idea of different goals when deciding for self versus other, Kray and Gonzales^8^ showed that in multi-attribute choice, people adapted a more uniform weighting function when deciding for self. When deciding for others, they paid attention to what is the most important factor. Kray^9^ later argued that advisers may adapt a different frame for self (“what do I want?”) versus other (“What would most people do?”).

Thus, findings about risk attitudes when deciding for others have not been consistent and various theories have been proposed to explain the inconsistencies in terms of differences in experimental paradigms or the decision goals within each paradigm when making decisions for oneself versus when for others. A much less studied but important hypothesis to account for the instability of risk attitudes for self and other is a motivational hypothesis. Previous research has suggested that emotions such as blame and guilt might be heavier burdens if the consequences of mistakes affect others, for example when we advise others. Tetlock^10^ showed that advisers (i.e. participants who suggest decisions for others) are burdened more heavily by accountability compared to decision makers for themselves. Similarly, Slovic^11^ showed that advisers (compared to personally involved decision makers) are more cautious about recommending a course of action than the task setup justified. Both raise the hypothesis that when deciding for others, the burden of anticipated regret will be greater.

One must exercise caution, however, when considering studies that looked at advice giving and aiming to draw predictions about decision making for others. Advice giving is, inherently, deeply connected to deliberative argumentation. The purpose of advice giving is often to make sense of a situation^12^. Proxy decision making, however, does not necessarily require providing any justification. In addition, when gambling with other people’s money, the proxy decision maker has no direct stake in the outcome. This could mean less stress and less worry about counterfactuals and regret. Indeed, recklessly betting with other people’s money without any accountability for the negative consequences of those bad investments has been argued as the root cause of the 2008 financial crisis^13^. It is, therefore, possible that decisions for others may be less affected by anticipated regret compared to decisions for self. Our study will test how anticipated regret impacts on risky decision making to benefit the self or to benefit another person.

## Audience effects

Numerous studies of social facilitation^14,15^ have shown that being in the presence of others changes our state of arousal^16^ and attention^17^ as well as altering valuation and decision making in a variety of situations^18^. More specifically, being watched may lead people to engage processes of self-presentation^19^ and reputation management^20^. These findings suggest that a variety of mechanisms may impact choosing for others if the beneficiary is present. For example, the participant’s state of arousal and/or selective attention may be affected by the presence of the audience. Alternatively, the decision maker may try to manage how the audience would judge them. This latter hypothesis is consistent with motivational theories of proxy decision making discussed above where accountability and justification are important^10,11^.

A number of previous studies reported that people tend to be more risk averse in the presence of an audience observing the decision maker’s behaviour^21,22^. The opposite tendency has also been reported in somewhat different experimental conditions, e.g. in mere physical presence of others who did not observe the decision maker’s behaviour^23^, and in adolescents^24^.

Very little is known about how anticipated regret may impact value-based decisions in the presence of an audience who can observe the agent’s choices and the outcomes. As demonstrated in our example of ordering wine for the table, understanding the impact of audience on regret and risk has profound implications for understanding everyday decision making and proxy decision making. Previous research that looked at regret in social contexts mainly focused on how other interacting agents (rather than a present, stake-holding audience) may affect the decision maker’s choices^25^ through socially expressed regret^26,27^, risky shifts^28^, or group polarization^29^. Here, we hypothesise that presence of others would accentuate the responsibility and reputation concerns for the decision maker under the scrutiny of the audience. Thus, we predicted that anticipated regret would drive choices more strongly in the presence of an audience.

In summary, we asked how two social contextual factors, deciding for self vs other and presence or absence of the other, modulate the role of anticipated regret, risk and expected value in decision making under outcome uncertainty. We adapted an experimental paradigm that has been widely used in behavioural economics^30–32^ and across various cultures^31^ to investigate the role of implicit, anticipated regret in value-based choices with uncertain outcomes. We manipulated risk and regret using a Wheel-of-Fortune task (Fig. 2), where the participant chose between a pair of gambles and received trial-by-trial factual and counterfactual feedback. Factual feedback indicated the outcome of gamble the participant chose to play. Counterfactual feedback indicated the outcome of the unchosen gamble and was provided to induce the emotion of anticipated regret at the time of making the choice. Risk was manipulated by changing the win/loss probability in the outcome combinations for the two alternative gambles presented in each trial. In this paradigm, we could model performance as a function of three parameters: difference in expected value between the two gambles, risk (i.e. variability of the possible outcomes) and the anticipated regret^31^.

**Figure 1:**
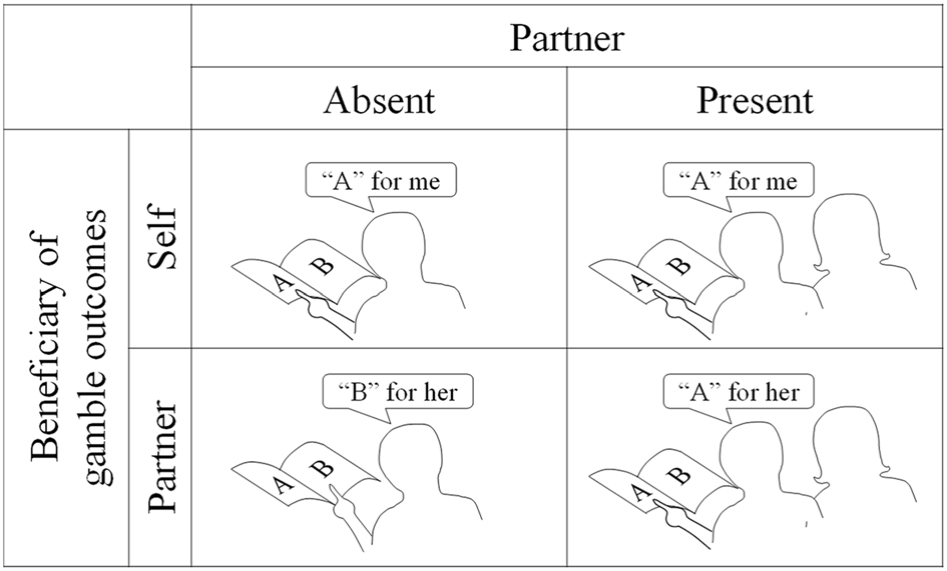
The 2 × 2 experimental design combined beneficiary (choice for self or partner) and audience (partner present or absent) conditions. Note that “self/absent” (the upper left) condition is the control condition adapted from previous studies^30^.

We manipulated the audience factor by having a passive partner (who was sometimes the beneficiary of the participant’s gambles) be present in or absent from the laboratory testing room. The participant and his/her partner were friends and were jointly recruited to attend the study together. Our study, therefore, consisted of a 2 × 2 design (Fig. 1) combining decision making for self (vs partner) with presence (vs absence) of the partner. Importantly, by testing the participants when they make decisions for themselves in the absence of the partner (Fig. 1, upper left cell), we tested our setup’s validity by replicating the findings from previous studies, namely that participants prefer options with higher expected value, less risk and less anticipated regret^30–32^.

## Methods

### Participants

A total of 50 healthy participant pairs, who had no history of mental or neurological disorders, took part after providing informed consent. Members of each pair were familiar with one another and recruited together. To determine the sample size, we analysed data we had previously gathered for a different study^31^ that used exactly the same gamble list (48 trials) and a procedure similar to our control (gambling for self, partner absent) condition. We used the SIMR package (version 1.0.3^33^) in R. The results suggested that we would have sufficient power (greater than 80%) to detect the regret effect at a false positive rate of 5% with a total sample of 50 participants (see Supporting Information for more details).

Among the 50 pairs, 5 were male–male pairs, 18 were male–female pairs, and 27 were female–female pairs. They were randomly assigned—by a coin toss—to the role of either gambling player (hereinafter called player) or observer (hereinafter called partner). Consequently, we tested 37 female and 13 male participants in the role of players, and 35 females and 15 males as partners. The age of the players was 23.4 ± 4.6 (mean ± standard deviation). The age of the partners was 23.3 ± 3.7. A two-tailed paired t test and Chi-squared test showed difference in neither age nor gender between players and partners [t(49) = 0.16, p = 0.876, and χ^2^(1) = 0.050, p = 0.824, respectively]. All experimental procedures were approved by the research ethics committee at University College London (ICN-AH-PWB-3-3-2016c) and were performed in accordance with the 1964 Declaration of Helsinki.

### Experimental design

We used variant of a Wheel-of-Fortune task^30,32^. Social context was controlled by a 2 × 2 design (Fig. 1) in which the beneficiary of gamble outcomes (player vs partner) and presence of the partner (present, absent) were manipulated. In half of the trials the player would be playing “for-self” where the outcome of the player’s choices determined her own earnings. In the other, “for-partner” half of trials, the player would be choosing on behalf of the partner. The partner’s earnings would depend on the outcome of the choices made by the player. In the “partner present” condition, the partner sat in the testing room (see Supporting Fig. S1) and watched the player make her choices sometimes on her own behalf and sometimes for the partner. In the partner-absent trials, players were tested alone in the experimental room. Meanwhile, the partner and experimenter waited outside of the testing room and did not receive any implicit or explicit information about their player’s decisions. In the partner-present trials, both the partner and the experimenter were present in the testing room.

The order of the conditions was randomized across participants and administrated by blocks. Blocks were separated by short rest intervals. Before each condition and after every 12 trials, a message was presented on the screen in the interval to remind the participants about the condition: “Gamble for SELF” or “Gamble for PARTNER.”

The player’s task was to choose which gamble s/he wanted to play, and respond to questions about her/his subjective states after trial outcomes. The partner was instructed to watch the player making choices and try to predict the player’s internal state. Specifically, they were asked to rate the player's feeling on a 7-point scale ranging from "extremely negative" to "extremely positive", which was a simplified version of the emotional rating task for players. This was a dummy task to encourage the player’s sense of being watched by the partner and motivate the partner's participation.

**Figure 2:**
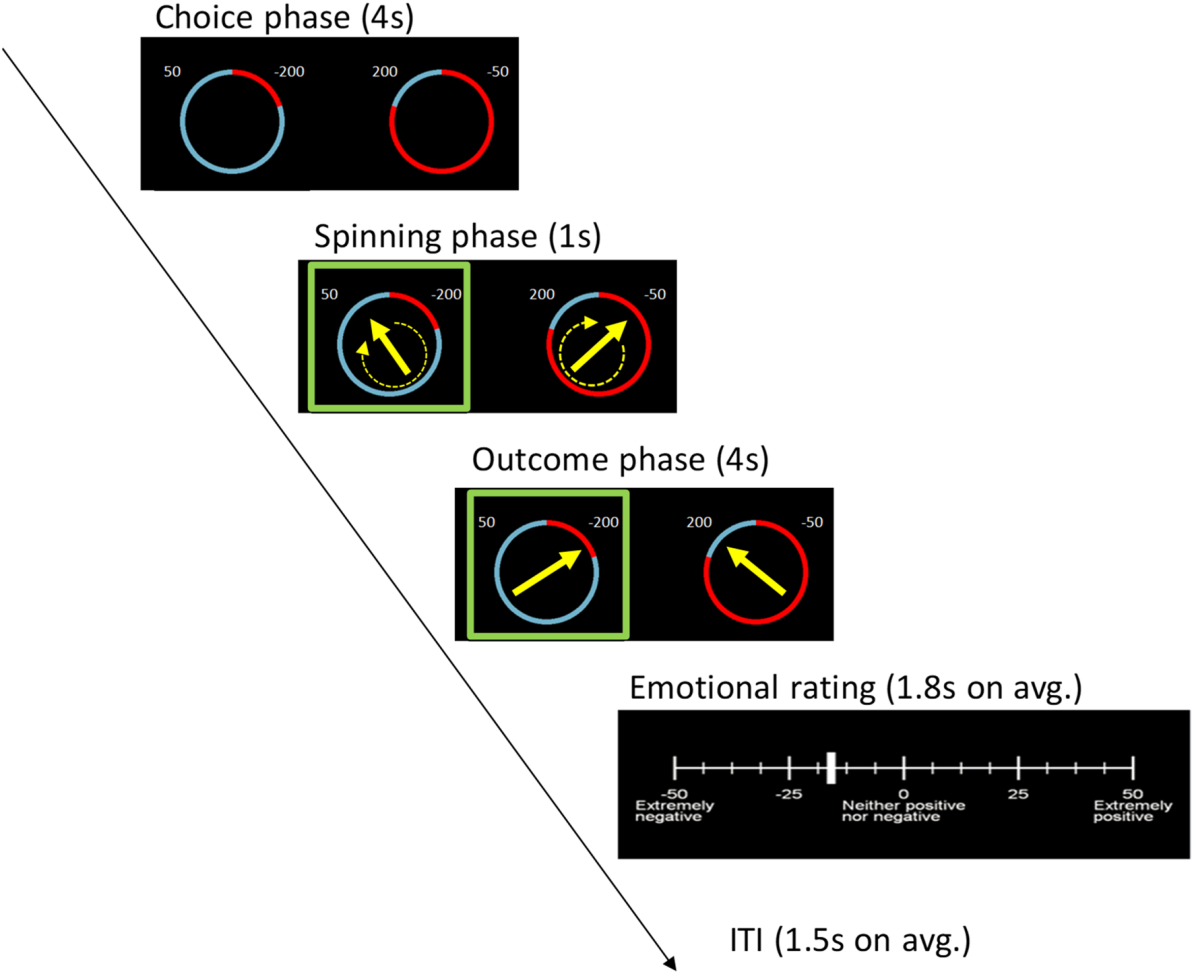
Timeline of events in a trial. Each trial started with two lotteries presented on the computer screen. The participant chose the lottery she wished to play (response window: 4 s). The selected lottery was highlighted with a green square. An arrow appeared in the centre of each lottery and began spinning. Outcomes were determined by the end position of the arrows. The end location of the arrow in the unchosen lottery indicated the counterfactual outcome. The participant then rated her feelings on a numerical scale of − 50 (extremely negative) to 50 (extremely positive).

In each trial (Fig. 2), the player evaluated two different lotteries and selected between them. Each lottery had two potential outcomes indicated numerically. The probability of each outcome was illustrated graphically by the size of an arc segment in a circle. Outcomes could be a gain or loss of points. The better outcome was always drawn in blue while the worse one in red: both could be either gain or loss. Each trial began with a 4 s choice period in which they viewed both options and chose by pressing a left or right button on keyboard. Once selected, the chosen gamble was highlighted in a coloured box frame. Then an arrow appeared in the centre of circle for each lottery (both the selected and unselected). After the 4 s choice period finished, the arrows span for 1 s and finished at a randomly-determined final location that indicated the outcome in each lottery. The outcome was displayed for 4 s, after which the participant rated their feeling about the outcome by adjusting a cursor using the left and right buttons on a number axis ranged from − 50 (“extremely negative”) to 50 (“extremely positive”); with the midpoint, zero, being “neither positive nor negative”.

By providing feedback about the unchosen gamble, our paradigm was inspired by previous literature^30,32^ to elicit regret and measure the impact of social context on this emotion. Here, regret is experienced when the outcome of chosen option is worse than the counterfactual outcome of the unchosen option.

All players performed a practice session with 12 trials in front of the partner. In the testing session they completed the full stimulus list in pseudorandom order, with each item presented once (i.e. 48 trials) for each condition. Total time for testing was 10.3 ± 0.5 min for each condition.

### Hardware settings

We used a laptop, SONY HDR-AS200V Action Cam (which captures 1920 × 1080 pixels images at 30 Hz with a size of 24.2 mm × 46.5 mm × 81.5 mm and a weight of 68 g), and a 23.8-inched extra monitor, Dell UltraSharp U2414H LED monitor (1920 × 1080 pixels at 60 Hz). Players used the laptop to play the gambles and rate their feelings. The camera captured the player’s face during the experiment which was then displayed live on the extra monitor for the partner to see. Additionally, this setup enables us to record the natural facial videos and to use them for later analyses on facial emotions. The extra monitor and partner seat were located behind the player’s seat by about 2 m, so that the player, participant and extra monitor were arranged in a triangle (see Supporting Fig. S1). This means the partner could watch both the face video on the monitor and the player’s laptop, while the player could watch only the laptop. Testing for players was conducted using Psychtoolbox in Matlab. Partners performed their task with paper and pencil.

### Payment

Participants were informed that their payment depended on the outcome of player’s decisions in the experiment. This consisted of fixed endowment (7.5 GBP per hour) and performance-based bonus for each individual. The bonus was the summation of the outcomes of two trials picked at random, one from partner-present and one from Partner-ABSENT conditions, for both player and partner, respectively. The value of each gamble was presented in points which were linearly converted to GBP (200, 50, − 50, and − 200 points were converted to 2.5, 1.5, 1.0 and 0 GBPs, respectively). The mean ± SD bonus across conditions was £1.57 ± 0.91.

### Behavioural modelling of choice

We performed a two-step analysis for choice behaviour. The first step is a mixed-effects logistic regression model, which was fit independently for each of the 2 × 2 conditions. The second step is a two-way repeated measures ANOVA using the obtained mixed-effects (each person’s) coefficients independently. Although less explored in the field of decision making, two-stage analyses are widely used for meta-analysis in various domains and models, e.g., hierarchical, mixed-effects models^34,35^.

Regarding the first step, our model-based analysis of participants’ choices sought to determine whether our decision variables of interest: risk and anticipated regret, influence choice and to quantify them for further analysis. Following previous works^30–32^, we evaluated performance in the four conditions by fitting a mixed-effect regression model separately for each condition, with the three regressors of interest. The first was the difference in the expected value (dEV) of the two gambles in a given trial. Our second regressor was the difference in variability of the outcomes, quantified by the difference in the gambles’ standard deviation (dSD, risk). The third was the anticipated regret (AR) which was defined as the difference in maximum possible experienced regret^30–32^. Experienced regret, in turn, is the difference between the factual and counterfactual outcome and can only be assessed after the outcome has occurred. As such, one might say anticipated regret corresponds to the difference between the best possible counterfactual outcome and the worse possible factual outcome. The mixed-effect regression coefficients obtained for each individual indicate the individual’s estimated weight of or preference for dEV, dSD and AR, respectively.

We estimated these model parameters for participants by using the glmer function in the R package lme4 (version 1.1–17)^36^. We modelled choice behaviour with a mixed-effects logistic regression model:

1 $${\text{logit}}\,\left( {{\text{Pr}}\,\left( {y_{i,t} = 1} \right)} \right) = x_{t} \beta + z_{t} {\mathbf{b}}_{i} + \varepsilon$$

2 $${\mathbf{b}}_i\sim {\mathcal{N}}(0,\sigma^{2} {\mathbf{S}}({{\varvec{\uptheta}}}))$$

3 $$\varepsilon \sim {\mathcal{N}}(0,\sigma^{2} {\mathbf{I}})$$

where *y* is the choice (1: left option, 0: right option), *i* denotes subject number, *i* = 1,2,…*N*, and *t* denotes the trial number. *x*_*t*_ and *z*_*t*_ are 1 × *l* and 1 × *m* row vectors comprising the rows of design matrices corresponding to *t*-th trial of *i*-th subject; *l* and *m* are the number of fixed- and random-effect variables, respectively. *β* and *b* are the model parameters to be estimated. *β *is an *l* –by-1 fixed-effect vector, and *b*_*i*_ is an *m*-by-1 random-effect vector. ${\mathcal{N}}$ refers to normal distribution. *S* is a symmetric and positive semidefinite matrix, parameterized by a variance component vector *θ*. We use a diagonal matrix for *S* assuming the independence of random-effect variables. **I** is an identity matrix, and *σ*^2^ is the error variance. The main assumption behind this model is that the coefficients of the fixed-effect variable *β* reflect the population level, while the random-effect variables *b* reflect the deviation from the population level for each subject. The random-effect coefficients *b*_*i*_ vary across subjects. The model parameters were estimated as best linear unbiased estimates (BLUE) for fixed effects and best linear unbiased predictions (BLUP) for random effects by using the glmer function in the R package lme4. We used the nloptwrap nonlinear optimizer for parameter learning, following^31^.

In our model, *x*_*t*_ and *z*_*t*_ are identical and consist of three gamble statistics: dEV, dSD, AR, explained below; none of their interactions was used in the present study. These arise as each trial, which consists of left and right lotteries, has six pseudo-independent parameters: two possible outcomes of the left lottery, *x*_*L*_ and *y*_*L*_ (*x*_*L*_ > *y*_*L*_); the probability of earning *x*_*L*_, *p*_*L*_ (which entails that the probability of *y*_*L*_ is 1 − *p*_*L*_); two possible outcomes of the right gamble, *x*_*R*_ and *y*_*R*_ (*x*_*R*_ > *y*_*R*_); and the probability of earning *x*_*R*_, *p*_*R*_ (the probability of *y*_*R*_ is 1 − *p*_*R*_).

dEV is the difference in expected value between the left and right gambles in a trial, namely:

4 $$dEV = EV_{L} - EV_{R} ,$$

where $EV = px + (1 - p)y$ for each option.

dSD is the risk factor, measured as difference in weighted standard deviation, namely:

5 $$dSD = SD_{R} - SD_{L} ,$$

where $SD = \sqrt {p(x - EV)^{2} + (1 - p)(y - EV)^{2} }$ for each gamble.

AR is an anticipated regret factor (regret factor in short, unless necessary) previously used by e.g.^30^, defined as:

6 $$AR = \;|y_{R} - x_{L} | - |y_{L} - x_{R} |$$

The second step of our two-step analysis is a two-way repeated measures ANOVA using the obtained mixed-effects (each person’s) coefficients of e (dEV), sd (SD) or r (AR) independently.

Moreover, we have also tested disappointment factor previously used by^30^ instead of and in addition to dSD. However, consistent with^31^, adding the disappointment factor did not improve the Bayesian information criterion (BIC) in our models. This is probably a reasonable result given that the way the task was implemented did not allow for the proper empirical separation of disappointment and regret.

### Modelling of emotional rating

We also measured *experienced* emotions at the end of each trial. This allowed us to ask if experienced emotions varied as a function of beneficiary and audience. Previous works investigating regret using a similar task found divergences between experienced and anticipated regret^30^. Whether regret-based choices and outcome ratings are in line is important for the interpretation of the results. To disentangle the impact of the beneficiary and audience on experienced emotions, we performed a two-stage analysis similar to that for the choice behaviour explained in previous section on emotional ratings.

In the first stage, similar to our choice behaviour analysis, emotional ratings were linear-regressed with objective measures of experienced emotions as mixed effects for each condition in the 2 × 2 design, testing the hypothesis that emotion ratings were negatively correlated with outcome-defined experienced emotions. Unlike the anticipated regret (Eq. 6), the hypothesis here depends on both the choice and the outcome. Following previous works^30^, we used experienced regret and disappointment factors as explanatory variables. The experienced regret factor was defined as the difference between the obtained outcome of unchosen gamble and the obtained outcome of chosen gamble. The experienced disappointment was defined as the difference between the unobtained and obtained value of the chosen gamble. For example, in Fig. 2, we see that the experienced regret factor in this specific trial is calculated as (200 − (− 200) = 400) and the experienced disappointment factor is given by (50 − (− 200) = 250). Based on previous works^30^, we reasoned that the full feedback design—where the outcomes of chosen and unchosen gambles are given—would be sufficient for estimating the contribution of regret and disappointment to the emotional ratings. This is because factual outcomes are sufficient for estimating the contribution of disappointment to ratings and they are indeed available in the full-feedback design.

In the second stage, the mixed-effects coefficients obtained in the first stage were compared across conditions. We tested if the effect of experienced regret and disappointment varied by our experimental design (Fig. 4).

### Statistical analysis

In addition to the analyses described above, statistical tests were carried out using the Wald test for logistic regression coefficients, repeated measures analyses of variance (ANOVA) in R statistical computing software (version 3.4.4)^37^. Reported p values are two-tailed.

## Results

### Choice behaviour

**Figure 3:**
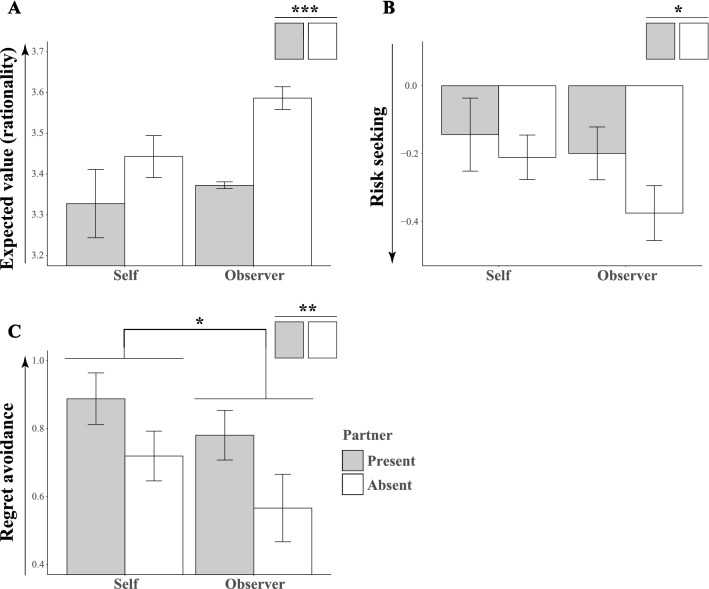
Regression analysis results (mixed-effects). Error bars show 95% confidence interval in the mixed effects found in the coefficients fitted to the individuals (n = 50). There was always a main effect of audience for all the three parameters. The main effect of the beneficiary was less consistent. There were no interactions between beneficiary and audience. (*p < 0.05; **p < 0.01; ***p < 0.001).

Main results are illustrated in Fig. 3 with corresponding statistics in Table S1. To facilitate comprehension, a labelled arrow to the left of each Y axis explains the psychological interpretation of each coefficient. The parameter estimates for expected value (Fig. 3A) and anticipated regret (Fig. 3C) were significantly different from zero in all the four conditions (z > 3.69, p < 0.001). These results mean that expected value and anticipated regret affected the participants’ decisions across all conditions. The impact of risk (variability; Fig. 3B) was less consistent: the effect was significant only when participants made a decision on behalf of the absent partner (z = − 2.70, p = 0.007) but not in the other three conditions (|z|< 1.66, p > 0.098). Importantly, the results from the control condition (“self/absent”, Fig. 1) were consistent with previous reports^30^ that lottery choices are driven by differences in expected value and an aversion to anticipated regret.

To disentangle the differential impact of the social influence and audience on decisions made for self vs others, we used the estimated mixed-effects parameters to draw parameter-wise comparisons between conditions. First, we examined the *difference in expected value* parameter (dEV, Fig. 3A, Table 1A) which indexes how much weight participants placed on whether one gamble was better than the other in terms of their expected value. A repeated measures ANOVA showed a main effect of audience (F_(1,49)_ = 17.47, *p* < 0.001, *η*^2^ = 0.263, 95% CI = [0.078, 0.447]). Choices were more strongly driven by difference in expected value when the partner was absent (Table 1A). There was a trend toward significance for the effect of beneficiary (F_(1,49)_ = 3.31, *p* = 0.075, *η*^2^ = 0.063 [0.000, 0.228]) meaning that the participants were slightly more likely to pursue the gamble with higher expected value when they chose for the partner, given the same difference in expected value. The interaction between audience and beneficiary was not significant (F_(1,49)_ = 1.30, *p* = 0.260, *η*^2^ = 0.026 [0.000, 0.165]).

**Table 1:** Results of repeated measures ANOVA for choice behaviour.

	DFn	DFd	SSn	SSd	F	p	*η*^2^ [95% CI]
**(A) Expected value (dEV) factor**							
(Intercept)	1	49	2356.38	10.74	10,746.75	< 0.001***	–
Beneficiary	1	49	0.443	6.56	3.31	0.075	0.063 [0.000, 0.228]
Partner	1	49	1.35	3.80	17.47	< 0.001***	0.263 [0.078, 0.447]
Beneficiary × partner	1	49	0.12	4.56	1.30	0.260	0.026 [0.000, 0.165]
**(B) Risk (dSD) factor**							
(Intercept)	1	49	10.78	45.43	11.63	0.0013**	–
Beneficiary	1	49	0.60	8.70	3.40	0.071	0.065 [0.000, 0.230]
Partner	1	49	0.74	8.17	4.42	0.041*	0.083 [0.000, 0.255]
Beneficiary × partner	1	49	0.15	7.21	1.01	0.320	0.020 [0.000, 0.152]
**(C) Anticipated regret (AR) factor**							
(Intercept)	1	49	109.06	36.81	145.18	< 0.001***	–
Beneficiary	1	49	0.85	10.29	4.03	0.050*	0.076 [0.000, 0.246]
Partner	1	49	1.83	10.43	8.61	0.0051**	0.149 [0.015, 0.335]
Beneficiary × partner	1	49	0.026	6.89	0.19	0.670	0.004 [0.000, 0.101]

Partner: partner presence. DFn and DFd: degrees of freedom in the numerator and denominator, respectively. SSn and SSd: sum of squares in the numerator and denominator, respectively.

Next, we examined the effect of the *risk* parameter (dSD, Fig. 3B). More negative parameter estimates would indicate higher risk-seeking in the participant choices. A repeated measures ANOVA on the dSD (risk) parameter showed a main effect of audience (F_(1,49)_ = 4.42, *p* = 0.041, *η*^2^ = 0.083 [0.000, 0.255]): participants were more strongly driven by bigger risks when their partners were absent (Table 1B). There was a trend toward significance for the main effect of beneficiary (F_(1,49)_ = 3.40, *p* = 0.071, *η*^2^ = 0.065 [0.000, 0.230]). Participants were more strongly driven by bigger risks when playing on behalf of their partners. The interaction between audience and beneficiary was not significant (F_(1,49)_ = 1.01, *p* = 0.320, *η*^2^ = 0.020 [0.000, 0.152]).

Finally, we examined the *anticipated regret* (AR, Fig. 3C) parameter. This parameter quantified the impact of anticipated regret (what counterfactual outcome might happen if unchosen option had been chosen) on decision making. Here, a large value of AR parameter would indicate a stronger impact of anticipated regret on the participant’s choices. A repeated measures ANOVA on AR showed a main effect of audience (F_(1,49)_ = 8.61, *p* = 0.0051, *η*^2^ = 0.149 [0.015, 0.335]). Participants were more cautious of anticipated regret when their partner was present (Table 1C). A main effect of beneficiary (F_(1,49)_ = 4.03, *p* = 0.050, *η*^2^ = 0.076 [0.000, 0.246]) was also observed: when the players gambled for the partner, they were less affected by concerns of anticipated-regret. There was no interaction between audience and beneficiary (F_(1,49)_ = 0.19, *p* = 0.670, *η*^2^ = 0.004 [0.000, 0.101]).

Moreover, a combined model that includes the interactions between conditions (using dummy regressors) and dEV, dSD and AR respectively only showed the main effects of dEV (‘e’, p < 0.001) and AR (‘r’, p < 0.001).

### Emotional ratings

We first focused on the baseline condition (decisions for self, partner absent) as a validity check to demonstrate that the ratings of experienced emotions made sense, e.g., were ratings more positive when the outcome was more pleasant? Consistent with our hypothesis and previous works^30,32,38^, we found that emotional ratings were negatively correlated with both experienced regret factor (*b* = − 0.25, df = 56.8, *p* < 0.001) and experienced disappointment factor (*b* = − 0.59, df = 60.8, *p* < 0.001).

**Figure 4:**
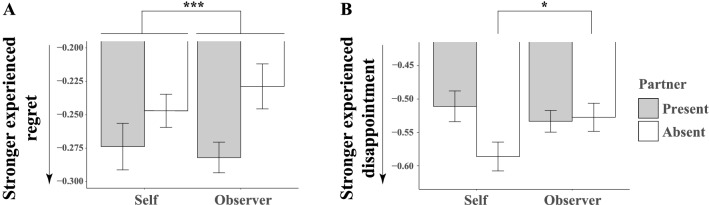
Experienced emotions were modulated by audience and beneficiary manipulations. This figure can be seen in a similar way to Fig. 3. Error bars show 95% confidence interval in the mixed effects found in the coefficients fitted to the individuals (n = 50).

A repeated measures ANOVA showed a very strong main effect of audience on experienced regret (F_(1,49)_ = 18.77, *p* < 0.001, *η*^2^ = 0.277 [0.088, 0.459]). The effect of beneficiary (F_(1,49)_ = 0.15, *p* = 0.702, *η*^2^ = 0.0030 [0.000, 0.096]) and their interaction between the two factors (F_(1,49)_ = 1.37, *p* = 0.247, *η*^2^ = 0.027 [0.000, 0.167]) did not reach significance. Stronger regret was expressed the partner was present (Fig. 4A, Table S3A) irrespective of whether the participant made for themselves or for their partner. In Fig. 4, note that the Y-axes corresponds to the (beta) regression coefficients driven from linear mixed models that test our two hypotheses and, importantly, not the emotional rating values themselves.

To examine the impact of the experimental factors on experienced disappointment (Fig. 4B), a repeated measures ANOVA was employed and showed a main effect of audience meaning that stronger disappointment was expressed when the partner was absent (F_(1,49)_ = 5.07, *p* < 0.029, *η*^2^ = 0.094 [0.000, 0.269]). A significant interaction between audience and beneficiary (F_(1,49)_ = 10.21, *p* = 0.0024, *η*^2^ = 0.172 [0.025, 0.359]) meant that strongest disappointment was expressed by participants when playing alone for themselves compared to the other three conditions (Fig. 4B, Table S3B). Post-hoc comparison for self vs partner, when audience was absent confirmed this impression F_(1,49)_ = 6.58, *p* = 0.013, *η*^2^ = 0.118 [0.005, 0.300], Table S.3B’). However, we would caution that our hypothesis did not make a specific prediction about this comparison.

Consistent with the previous literature^39^, there was some overlap between the results from anticipated and experienced regret and there was also some areas of divergence. The strong effect of audience was common to both measures. The effect of beneficiary which was less strongly observed for anticipated regret, was not observed for experienced regret. We return to this point in “Discussion”.

A combined model that includes the interactions between conditions (using dummy regressors) and experienced regret and disappointment respectively failed to converge, so it could not be analysed.

## Discussion

We set out to investigate the role of two social factors on anticipated regret in risky, value-based decision making with uncertain outcomes. Using a 2 × 2 design we measured the impact of beneficiary (outcome for oneself or for another, familiar person) and audience (whether the familiar other was present and could witness the decisions and outcomes or not). By having participants choose between probabilistic lotteries and providing factual and counterfactual outcomes, we measured the participants’ attitude towards risk, expected value and anticipated regret under the four combinations of our experimental design. Most prominently, we found a robust impact of audience on all these factors. In the presence of a familiar other, participants were more risk- and regret averse and less likely to choose the lottery with the higher expected value. The beneficiary effect was less consistent and observed most strongly in higher anticipated regret when participants made decisions for themselves.

Our main hypothesis was that the two social manipulations (beneficiary and audience) change the burden of responsibility for the decision maker. In order to examine this hypothesis, we focused on measuring the impact of regret on risky decisions. Regret is a negative emotion that arises when participants contemplate the counterfactual that a better outcome would have been achieved if they had made a better choice. Anticipated regret arises when, at the time of a decision, participants imagine how much regret they might feel if they obtained a less than perfect outcome. This type of pre-choice counterfactual thinking can have a robust effect on choice behaviour^40–42^. The impact of anticipated regret on human decision making at individual^30,38,43,44^ and group level^26,28,29^ has been extensively studied. In the present study, we saw that individual decisions in the absence of others (upper left cell in Fig. 1) were driven by both anticipated regret and the expected value of the two gamble, thus replicating the key findings of previous works.

There is a long history of studies showing that people perform differently and make different decisions in the presence of an audience compared to when they are alone^15,19,45^. Previous works have shown that social facilitation causes people to act faster in the presence of a conspecific and is believed to be a consequence of general changes of arousal^15^. A specific audience effect, on the other hand, arises when participants believe they are being watched and evaluated by another person and change their behaviour to maintain a good reputation^19,20^. Our study taps into both of these mechanisms: the participant was conspicuously observed by the partner and experimenter in the Partner Present conditions but left to his/her own devices under Partner Absent condition. Moreover, the participant was under increased evaluative pressure when s/he gambled with the partner’s money in the Gamble for Partner conditions. Thus, both social facilitation/arousal and the need for reputation management were manipulated between the conditions to see how they might impact regret aversion.

The results showed a robust effect of the audience on all aspects of decision making that we measured. When observed by a partner, participants made more conservative choices—they chose the less risky option, and aimed to avoid regret but at the expense of a lower expected value. Being observed by the partner augmented counterfactual thinking and promoted the imagination of the regret that may follow leading to more conservative, defensive choices. This means that decisions when being watched were more likely to prevent the worst possible outcome compared to when deciding alone. This result is consistent with our hypothesis that the presence of an audience increases the burden of responsibility for the decision maker.

This is consistent with a recent meta-analysis linking the audience effect to social anxiety^46^. People who feel more social anxiety tend to show a larger and more negative audience effect (i.e. worse performance in the presence of others), while those without social anxiety may show a positive audience effect blooming under pressure in the presence of others. Whether social anxiety is directly connected to anticipated regret, or whether this connection is mediated by the presence of an evaluating audience is an important and useful question for future research. This question could be addressed by employing the design adopted here and concomitantly collecting explicit (e.g. questionnaires) or implicit (e.g. skin conductance response) measures of social anxiety.

In our study, participants could earn money for themselves or the other person in different blocks. A hypothesis drawn from earlier literature of advice giving^10,11^ predicted that anticipated regret should increase when making decision for others. The results (Fig. 3c), however, rejected this hypothesis. Instead they indicated that negative outcomes for others may have a reduced impact on the decision maker’s sense of regret.

Numerous studies have examined the impact of proxy decision masking on risk attitude and the findings have been inconsistent and contradictory (Ferrer et al.^3^). In keeping with that pattern, we also observed marginal, weak effects of beneficiary for expected value and risk, with trends towards participants choosing higher expected value and taking better, more measured risks when choosing for the partner. Thus, with this statistical caveat in mind, we prefer to avoid strong interpretations of our findings about attitudes to risk in proxy decision making.

Earlier research has suggested that people may choose to avoid different types of errors (c.f., omission vs commission errors described in introduction). Although those studies were indirectly instructive in helping us develop a set of contrasting hypotheses regarding the role of risk in proxy decisions, there is no straightforward mapping between outcome alternatives in our wheel-of-fortune design and omission vs commission errors. Therefore, whether our findings could be due to any difference between choice goals when deciding for self and other remains an open question.

Our findings are also consistent with previous studies in social problem solving where it has been demonstrated that people manifest inferior reasoning ability when asked to think of a social problem (e.g. what to do with a cheating partner?) in first-person point of view compared evaluating the same problem from third-person point of view. People were more aware of the limits of their own knowledge, made a bigger effort to find a compromise, and recognized the importance of factors outside of their control better when taking third-person’s perspective as well as when deliberately distancing themselves from the problem^1^. More recent studies demonstrated a positive correlation between these hallmarks of wise reasoning with emotion regulation^47^ and a negative correlation with the frequency of feeling both positive and negative emotions^48^. Interpreting our findings in the light of these studies suggests that when choosing for others, people may relinquish their first-person point of view and instead adopt their partner’s perspective. This change of viewpoint may facilitate emotional regulation of risk and regret and enable them to make more advantageous decisions.

It is important to note that the vast majority of previous studies that examined vicarious choice involved participants making choices for anonymous strangers. In our study, participants were deciding for themselves or for a friend (sometimes a romantic partner) and felt a close self-other overlap with their experimental partner (see Supporting Information). In this context, reputation costs for bad choices might be high but perhaps the distinction between rewards for self and other might be less salient: participants might have anticipated the possibility of sharing their earnings with their partner at the end of the experiment. Thus, our findings of strong effects of an audience and less pronounced effects of beneficiary should be interpreted with the impact of familiarity in mind.

Indeed previous research has shown that eye gaze is modulated by presence of an audience^49^ and gaze patterns are predictive of value-based decisions^50^. It is therefore very likely that our two experimental factors (Audience and Proxy) affect the allocation of spatial attention and eye gaze. Future research combining social factors and attentional measurements would be able to clarify such interactions.

Our empirical operationalization of regret, which has been used extensively in the literature of decision making is likely to have some limitations. Most notably, the subjective feeling of anticipated regret may be nonlinearly connected to the maximum difference between factual and counterfactual outcome. Such nonlinear relation is likely to be best captured by a sigmoid function. For the purposes of our study, we feel confident that this empirical operationalization was adequate. This is because our main findings (Fig. 3C) indicated that anticipated regret was modulated by both of our experimental manipulation of Audience and Proxy and therefore design was unlikely to have been afflicted by floor or ceiling effects.

We exactly followed the gamble table used in^30^ that includes both positive and negative points. However, regarding the payment, we employed a bonus system to ensure the minimum amount from an ethical point of view. However, choice behaviour is known to be very different in the gain and loss domains, e.g. loss aversion. Therefore, the differences of our results from some previous studies may stem from the different payment system. Furthermore, the way anticipated regret is calculated in the current study may include the counterpart, i.e. anticipated rejoicing (the emotion that occurs when the decider realizes the counterfactual outcome was worse than the obtained outcome)^32^, although, in the context of these types of decision making tasks, most of the emphasis in the literature has been on interpreting this type of factor in terms of regret. Moreover, the use of lottery design may constrain the generality of the finding^51^. Perhaps future studies can consider risky choice in health-medical settings, to test the generality of the findings to different settings.

We found an effect of disappointment on emotional rating, but not on choice behaviour in line with previous literature^30^. Interestingly, there was a strong correlation between the experienced regret factor and experienced disappointment factor (r = 0.70), which was different from the much weaker correlation between anticipated regret and disappointment factors (r = − 0.25; see^31^ for more details on this). On the one hand, it is possible that our observation is at least partially driven by the collinearity of the factors. On the other hand, these findings are in line with previous works on affective forecasting that showed a stronger correlation between experienced outcome and emotion compare to the correlation between anticipated outcome and emotion^52^. Although not straightforward in the specific case of the wheel-of-fortune paradigm, an important direction in future research would be to consider gamble parameters that better disentangle the emotional factors such as regret and disappointment as well as temporal factors such as the anticipated and experienced emotions.

In the present study, we opted for a two-stage analysis rather than a single-stage analysis. Two-stage analyses are widely used and have been compared to one-stage analyses for meta-analysis in various domains and models, e.g., hierarchical models. In short, one- and two-stage analyses may yield either similar or different results^34^. Although one-stage analysis uses a more exact likelihood specification and is therefore theoretically preferred, it often suffers from computational difficulties and convergence problems and is more prone to type-II errors^34,35^. Our results are generally consistent with these properties: the two-stage analysis proved to be more sensitive and the one-stage analysis suffered from convergence problems. These considerations became even more relevant when we tried to extend our model by adding other variables, such as the effect of time (e.g.^30^). Therefore, we caution the reader to be wary of these statistical issues when evaluating experimental paradigms to determine which type of analysis may be more suitable.

Finally, we note that the effect of beneficiary went in the opposite direction to the audience effect. On one hand, both previous data and our results suggest that when watched, people decide more conservatively. On the other hand, when choosing for others, participants were less constrained by anticipated regret on behalf of the partner. These results suggest that participants’ financial and reputational interests impacted the feeling regret independently. Participants were more concerned with regret when their own financial interest was at stake^13^. However, they also showed increased regret when their reputational interest was at stake in the audience present (vs absent) comparison. It is important to note the non-trivial nature of these findings. One might have expected that making decisions for others in their presence may have be the most emotionally challenging condition. However, our results are consistent with the notion that, across the experiment, emotional impact of regret on choices were driven primarily by self-interest albeit along the two orthogonal dimensions of monetary and social capital. It was amusing for us to observe that the participants were most strongly affected by the negative emotions of anticipated regret when they decided for themselves in the presence of the observing partner. This leads to perhaps the curious but useful recommendation that when friends want to have fun gambling at a casino, they should gamble with one another’s money and go to separate tables.

## Supplementary Information

Supplementary Information 1.

## Data Availability

The datasets generated during and/or analysed during the current study are available at https://osf.io/7wyfk/.
