# Supplementary material for: The role of anticipated regret in choosing for others
Source: Sci Rep. 2021 Jun 15;11:12557. doi: 10.1038/s41598-021-91635-z (PMC8206101; doi:10.1038/s41598-021-91635-z)
Supplement: Supplementary file 1 — Supplementary Information 1. [file 41598_2021_91635_MOESM1_ESM.docx]

# Supporting Information

The role of anticipated regret in choosing for others

Running head: regret in choosing for others

# Shiro Kumano^1,2*^, Antonia Hamilton^1^, Bahador Bahrami^3,4,5^

*SK: corresponding author, kumano@ieee.org

^1^ Institute of Cognitive Neuroscience, University College London; Alexandra House, 17 Queen Square, London, WC1N 3AZ, United Kingdom

^2^ NTT Communication Science Laboratories, Nippon Telegraph and Telephone Corporation; 3-1 Morinosato-Wakamiya, Atsugi, Kanagawa, 243-0198, Japan

^3^ Faculty of Psychology and Educational Sciences, Ludwig Maximilian University; Leopoldstrasse 13, 80802 Munich, Germany

^4^ Department of Psychology, Royal Holloway University of London; Egham, Surrey, TW20 0EX, United Kingdom

^5^ Centre for Adaptive Rationality, Max Planck Institute for Human Development, Lentzeallee 94, 14197 Berlin, Germany

**Experimental setups**

Observer

Experimenter

≈ 2m

Extra monitor

Player

≈ 1.7m

≈ 1.2m

Door

Video via HDMI cable

Laptop

Sound via audio cable

Camera

*Figure S.1 Device arrangement and cable connection (top view)*

*The camera captured the player’s face during the experiment which was then live on the extra monitor for the observer to see. The captured images were simultaneously combined with sound from the gambling laptop for synchronization and were recorded on a SD card in the camera. The extra monitor and observer seat were located behind player seat so that observers could watch both monitors while players could watch only the laptop. The experimenter sat on the floor at the white circle above in order not to be captured by the camera.*

# Methods

## Power analysis

To determine the sample size, we did a power analysis using data we had previously collected for a different study (Li Li et al., under review) using the same experimental paradigm as the control condition in our design. Consistent with ^1^, the results had showed a regret effect (N = 57, estimated regret coefficient = 0.0030, Z = 3.38, p < .001). Using SIMR package (version 1.0.3; ^2^), which estimates power based on Monte Carlo simulations, the data showed 92.90% power (C.I. = 91.13 - 94.41) with all participants (N = 57) to detect the regret effect at a false positive rate of 5%. To estimate the power for smaller sample size, we then generated 66 permutation groups of 50 people by dropping randomly selected 7 participants, and calculated the power for each group. The mean CI was 88.8%, and the mean CI lower bound (as a more rigorous criteria) was 86.8%. Therefore, we concluded that N = 50 is sufficient to detect the regret effect.

## Statistical analysis

We performed the following two-step analysis.

For choice behaviour:

Step 1: Fit a logistic regression, “choice ~ e + sd + r + (1|subj) + (0+e|subj) + (0 + sd|subj) + (0 + r|subj)”, where e, sd and r mean dEV, SD and AR, independently for 2x2 conditions using glmer function in the R package lme4^3,4^.

Step 2: Two-way repeated measures ANOVA using the obtained random (each person’s) coefficients of e (dEV), sd (SD) or r (AR) independently.

Moreover, we repeated Step 1 300 times by randomly changing the data order and used the best AIC model as the result. This is because we observed that the fitting results of ‘glmer’ function of lme4 package depend on the data order (i.e. different results were obtained when the data were randomly ordered). This suggests the difficulty of fitting mixed effects models. Therefore, it wouldn’t be a good idea to fit more complex models, such as unified models using all variables together (with conditions as dummy variables).

For emotional rating:

Step 1: Fit a linear regression, ratingZ ~ expRegretZ + expDisappointmentZ + (0+expRegretZ|subj) + (0+expDisappointmentZ|subj), where ratingZ, expRegretZ and expDisappointmentZ mean person-wise standardized emotional rating scores, standardized experienced regret (outcome of unchosen gamble minus outcome of chosen gamble) and standardized experienced disappointment (unrealized result minus outcome of chosen gamble).

Step 2: same as that for choice behaviour

Moreover, a combined model would be for choice behaviour:

choice ~ e*forWhom*withObs + sd*forWhom*withObs + r*forWhom*withObs + (1|subj) + (0+ e*forWhom*withObs |subj) + (0+ sd*forWhom*withObs |subj) + (0+ r*forWhom*withObs |subj)

This only showed the main effects of dEV (‘e’ , p < .001) and AR (‘r’, p < .001).

For emotional rating:

ratingZ ~ e*forWhom*withObs + sd*forWhom*withObs + r*forWhom*withObs + (1|subj) + (0+e*forWhom*withObs|subj) + (0+sd*forWhom*withObs|subj) + (0+r*forWhom*withObs|subj)

This took long time and didn’t converge.

## Pair relationship measure

The Inclusion of Other in the Self scale (IOS) ^5^ was used immediately after the experiment to measure the interpersonal closeness within each pair. This is a 7 point scale where participants select a pair of partly overlapping circles as a representation of their relationship with another person. It is believed to tap a sense of interpersonal closeness. The player and partner in each pair were informed that their answers would be never shared with each other, and then answered it separately.

# Results

## Close interpersonal relationship

To check how close participant’s felt to their partner in the task (who could be a friend or a romantic partner), we used the Inclusion of Other in the Self scale (IOS) ^5^. The mean score of given by players was 5.0 +/- 1.6 (standard deviation), while that given by partners was 4.8 +/- 1.4. This suggests that the pairs were on average in mutual agreement about the depth of their relationship, such as relations with romantic partners, close friends or family members ^6^. A paired t-test revealed no difference in IOS scores between players and partners (t(49) = 0.70, p = .49).

## No difference in number of missed trials, obtained outcomes, and RTs between conditions

The number of missed trials, namely the number of times in which no choice was made during the 4-sec choice phase, was 0.24 +/- 0.08 (standard error) on “for-self”-”partner-present” condition, 0.16 +/- 0.05 on “for-self”-”partner-absent” condition, 0.20 +/- 0.08 on “for-partner”-”partner-present” condition, and 0.20 +/- 0.08 on “for-partner”-”partner-absent” condition. A repeated measures ANOVA revealed no main effect of beneficiary (F_(1,49)_ = 0.0, *p* = 1.00), no main effect of audience (F_(1,49)_ = 0.329, *p* = .57) and no interaction (F_(1,49)_ = 0.395, *p* = .53).

The mean obtained outcome across the 48 trials was 53.4 +/- 2.2 gamble points on “for-self”-”partner-present” condition, 57.4 +/- 2.8 on “for-self”-”partner-absent” condition, 55.1 +/- 2.6 on “for-partner”-”partner-present” condition, and 59.6 +/- 2.3 on “for-partner”-”partner-absent” condition. A repeated measures ANOVA revealed no main effect of beneficiary (F_(1,49)_ = 0.58, *p* = .45), no main effect of audience (F_(1,49)_ = 2.41, *p* = .13) and no interaction (F_(1,49)_ = 0.009, *p* = .93).

The mean response time (RT) across the 48 trials was 1,909 +/- 55 msec on “for-self”-”partner-present” condition, 1,901 +/- 54 msec on “for-self”-”partner-absent” condition, 1,902 +/- 58 msec on “for-partner”-”partner-present” condition, and 1,871 +/- 61 msec on “for-partner”-”partner-absent” condition. A repeated measures ANOVA revealed no main effect of beneficiary (F_(1,49)_ = 0.37, *p* = .55), no main effect of audience (F_(1,49)_ = 0.65, *p* = .43) and no interaction (F_(1,49)_ = 0.18, *p* = .68).

## [Choice behaviour] Results of logistic regression

*Table S.1 Results of logistic regression (fixed effects) for both unstandardized (raw, in parentheses) and standardized explanatory variables, i.e. dEV (the difference in expected value between the left and right gambles), dSD (the risk factor, measured as difference in weighted standard deviation) and AR (anticipated regret factor); see the manuscript for more details about the variables. These were obtained the best results regarding AIC in 300 random repetitions with different data order to compensate small fluctuation in results. Their standard errors were small enough (<0.1%). The results of unstandardized and standardized explanatory variables were comparable. Therefore, we focused on the standardized results for the latter analyses.*

*(a) “for-self”-”observer-present” condition*

|  | Estimate | Std. err | Z | p |
| --- | --- | --- | --- | --- |
| (Intercept) | 0.117  (0.110) | 0.015  (0.016) | 1.329  (1.250) | .184  (.211) |
| dEV | 3.328  (0.025) | 0.084  (0.001) | 14.748  (14.772) | < .001***  (< .001***) |
| dSD | -0.144  (-0.0020) | 0.107  (0.0015) | -0.855  (-0.858) | .392  (.391) |
| AR | 0.888  (0.0045) | 0.076  (0.0004) | 5.788  (5.800) | < .001***  (< .001***) |

*(b) “for-self”-”observer-absent” condition*

|  | Estimate | Std. err | Z | p |
| --- | --- | --- | --- | --- |
| (Intercept) | 0.154  (0.142) | 0.000  (0.000) | 1.953  (1.809) | .0508  (.070) |
| dEV | 3.443  (0.026) | 0.051  (0.000) | 16.661  (16.679) | < .001***  < .001*** |
| dSD | -0.211  (-0.0029) | 0.065  (0.0009) | -1.655  (-1.660) | .098  (.097) |
| AR | 0.719  (0.0036) | 0.073  (0.0004) | 4.951  (4.942) | < .001***  (< .001***) |

*(c) “for-observer”-”observer-present” condition*

|  | Estimate | Std. err | Z | p |
| --- | --- | --- | --- | --- |
| (Intercept) | 0.117  (0.106) | 0.002  (0.002) | 1.467  (1.336) | .142  (.182) |
| dEV | 3.373  (0.026) | 0.008  (0.000) | 17.916  (17.904) | < .001***  (< .001***) |
| dSD | -0.199  (-0.0028) | 0.077  (0.0011) | -1.443  (-1.450) | .149  (.147) |
| AR | 0.781  (0.0040) | 0.073  (0.0004) | 5.264  (5.263) | < .001***  (< .001***) |

*(d) “for-observer”-”observer-absent” condition*

|  | Estimate | Std. err | Z | p |
| --- | --- | --- | --- | --- |
| (Intercept) | 0.140  (0.125) | .010  (0.011) | 1.650  (1.462) | .099  (.144) |
| dEV | 3.586  (0.027) | 0.028  (0.000) | 17.455  (17.412) | < .001***  (< .001***) |
| dSD | -0.375  (-0.0052) | 0.081  (-0.0011) | -2.701  (-2.710) | .0069**  (.0067**) |
| AR | 0.566  (0.0029) | 0.099  (0.0005) | 3.688  (3.693) | < .001***  (< .001***) |

## [Emotional rating] Linear regression

Formula: ratingZ ~ expRegretZ + expDisappointmentZ + (0 + expRegretZ | subj) + (0 + expDisappointmentZ | subj)

*Table S.2 Results of linear regression for standardized explanatory variables (i.e. experienced regret and disappointment).*

*(a) “for-self”-”observer-present” condition*

|  | Estimate | Std. err | df | t | p |
| --- | --- | --- | --- | --- | --- |
| (Intercept) | -0.0322 | 0.000 | 2312.46 | -2.677 | .00749** |
| expR | -0.274 | 0.0175 | 53.60 | -10.055 | < .001*** |
| expDisp | -0.511 | 0.0230 | 53.19 | -16.283 | < .001*** |

*(b) “for-self”-”observer-absent” condition*

|  | Estimate | Std. err | df | t | p |
| --- | --- | --- | --- | --- | --- |
| (Intercept) | -0.0336 | 0.000 | 2340.00 | 2.453 | .0142* |
| expR | -0.247 | 0.0124 | 56.84 | -9.403 | < .001*** |
| expDisp | -0.586 | 0.0215 | 60.75 | -18.364 | < .001*** |

*(c) “for-observer”-”observer-present” condition*

|  | Estimate | Std. err | df | t | p |
| --- | --- | --- | --- | --- | --- |
| (Intercept) | -0.0127 | 0.000 | 2342.09 | 0.95 | .342 |
| expR | -0.282 | 0.0115 | 62.90 | -11.28 | < .001*** |
| expDisp | -0.533 | 0.0162 | 67.05 | -19.10 | < .001*** |

*(d) “for-observer”-”observer-absent” condition*

|  | Estimate | Std. err | df | t | p |
| --- | --- | --- | --- | --- | --- |
| (Intercept) | -0.000854 | 0.000 | 2325 | -0.065 | .948 |
| expR | -0.229 | 0.0168 | 58.33 | -8.137 | < .001*** |
| expDisp | -0.527 | 0.0210 | 56.42 | -16.986 | < .001*** |

## [Emotional rating] Results of repeated measures ANOVA

*Table S.3 Results of repeated measures ANOVA*

*(A) Experienced regret parameter*

|  | DFn | DFd | SSn | SSd | F | p | *η*^2^ |
| --- | --- | --- | --- | --- | --- | --- | --- |
| (Intercept) | 1 | 49 | 13.31 | 1.191 | 547.51 | <.001*** | - |
| Beneficiary | 1 | 49 | 0.00127 | 0.419 | 0.148 | .702 | .00301  [.000, .096] |
| Partner | 1 | 49 | 0.0802 | 0.209 | 18.77 | <.001*** | .277  [.088, .459] |
| Beneficiary * Partner | 1 | 49 | 0.00877 | 0.313 | 1.370 | .247 | .0272  [.000, .167] |

*(B) Experienced disappointment parameter*

|  | DFn | DFd | SSn | SSd | F | p | *η*^2^ |
| --- | --- | --- | --- | --- | --- | --- | --- |
| (Intercept) | 1 | 49 | 58.20 | 2.325 | 1226.71 | <.001*** | - |
| Beneficiary | 1 | 49 | 0.0164 | 0.862 | 0.931 | .339 | .0186  [.000, .149] |
| Partner | 1 | 49 | 0.0598 | 0.578 | 5.070 | .0289* | .0938  [.000, .269] |
| Beneficiary * Partner | 1 | 49 | 0.0822 | 0.394 | 10.211 | .00244** | .172  [.025, .359] |

(B)’ Post-hoc single main effect analysis

(For self) audience present vs absent

|  | DFn | DFd | SSn | SSd | F | p | *η*^2^ |
| --- | --- | --- | --- | --- | --- | --- | --- |
| (Intercept) | 1 | 49 | 30.08 | 1.880 | 783.92 | <.001*** | - |
| Partner | 1 | 49 | 0.141 | 0.549 | 12.59 | <.001*** | .204  [.041, .391] |

(For observer) audience present vs absent

|  | DFn | DFd | SSn | SSd | F | p | *η*^2^ |
| --- | --- | --- | --- | --- | --- | --- | --- |
| (Intercept) | 1 | 49 | 28.13 | 1.306 | 1055.27 | <.001*** | - |
| Partner | 1 | 49 | 8.89e-04 | 0.422 | 0.103 | .750 | .002  [.000, .089] |

(Audience present) for self vs observer

|  | DFn | DFd | SSn | SSd | F | p | *η*^2^ |
| --- | --- | --- | --- | --- | --- | --- | --- |
| (Intercept) | 1 | 49 | 27.27 | 1.323 | 1009.94 | <.001*** | - |
| Beneficiary | 1 | 49 | 0.0126 | 0.617 | 1.000 | .322 | .020  [.000, .152] |

(Audience absent) for self vs observer

|  | DFn | DFd | SSn | SSd | F | p | *η*^2^ |
| --- | --- | --- | --- | --- | --- | --- | --- |
| (Intercept) | 1 | 49 | 30.99 | 1.579 | 961.58 | <.001*** | - |
| Beneficiary | 1 | 49 | 0.0859 | 0.640 | 6.583 | .0134* | .118  [.005, .300] |

# References

1 Coricelli, G. *et al.* Regret and its avoidance: a neuroimaging study of choice behavior. *Nature neuroscience* **8**, 1255-1262, doi:10.1038/nn1514 (2005).

2 Green, P. & MacLeod, C. J. SIMR: anR package for power analysis of generalized linearmixed models by simulation. *Methods in Ecology and Evolution* **7**, 493-498 (2016).

3 Bates, D., Maechler, M., Bolker, B. & Walker, S. Fitting Linear Mixed-Effects Models Using lme4. *Journal of Statistical Software* **67**, 1-48 (2015).

4 R Core Team. *R: A Language and Environment for Statistical Computing*. (R Foundation for Statistical Computing, 2020).

5 Aron, A., Aron, E. N. & Smollan, D. Inclusion of Other in the Self Scale and the structure of interpersonal closeness. *Journal of Personality and Social Psychology* **63**, 596-612, doi:10.1037/0022-3514.63.4.596 (1992).

6 Gachter, S., Starmer, C. & Tufano, F. Measuring the Closeness of Relationships: A Comprehensive Evaluation of the 'Inclusion of the Other in the Self' Scale. *PLoS One* **10**, e0129478, doi:10.1371/journal.pone.0129478 (2015).
